# Supplementary material for: Development of Two-Tube Loop-Mediated Isothermal Amplification Assay for Differential Diagnosis of Plasmodium falciparum and Plasmodium vivax and Its Comparison with Loopamp™ Malaria
Source: Diagnostics (Basel). 2021 Sep 16;11(9):1689. doi: 10.3390/diagnostics11091689 (PMC8467429; doi:10.3390/diagnostics11091689)

Table S1: Cross-tabulation of index tests (Visual LAMP and Loopamp^{TM}) results by the results of reference standard test (Nested PCR) for the diagnosis of malaria (*P. falciparum, P. vivax* and *Plasmodium* genus).

Case no.	Nested PCR		Two tube Visual LAMP[@]		LoopampTM		
	Pv	Pf	Pv	Pf	Pan[#]	Pf[$]	Pv^
1.	Positive	Negative	Positive	Negative	Positive	Negative	Positive
2.	Positive	Negative	Positive	Negative	Positive	Negative	Positive
3.	Positive	Negative	Positive	Negative	Positive	Negative	Positive
4.	Positive	Negative	Positive	Negative	Positive	Negative	Positive
5.	Positive	Negative	Positive	Negative	Positive	Negative	Positive
6.	Positive	Negative	Positive	Negative	Positive	Negative	Positive
7.	Positive	Negative	Positive	Negative	Positive	Negative	Positive
8.	Positive	Negative	Positive	Negative	Positive	Negative	Positive
9.	Positive	Negative	Positive	Negative	Positive	Negative	Positive
10.	Positive	Negative	Positive	Negative	Positive	Negative	Positive
11.	Positive	Negative	Positive	Negative	Positive	Negative	Positive
12.	Positive	Negative	Positive	Negative	Positive	Negative	Positive
13.	Positive	Negative	Positive	Negative	Positive	Negative	Positive
14.	Positive	Negative	Positive	Negative	Positive	Negative	Positive
15.	Positive	Negative	Positive	Negative	Positive	Negative	Positive
16.	Negative	Positive	Negative	Positive	Positive	Positive	Negative
17.	Negative	Positive	Negative	Positive	Positive	Positive	Negative
18.	Negative	Positive	Negative	Positive	Positive	Positive	Negative
19.	Negative	Positive	Negative	Positive	Positive	Positive	Negative
20.	Negative	Positive	Negative	Positive	Positive	Positive	Negative
21.	Negative	Positive	Negative	Positive	Positive	Positive	Negative
22.	Negative	Positive	Negative	Positive	Positive	Positive	Negative
23.	Negative	Positive	Negative	Positive	Positive	Positive	Negative
24.	Negative	Positive	Negative	Positive	Positive	Positive	Negative
25.	Negative	Positive	Negative	Positive	Positive	Positive	Negative
26.	Negative	Positive	Negative	Positive	Positive	Positive	Negative
27.	Negative	Positive	Negative	Positive	Positive	Positive	Negative
28.	Negative	Positive	Negative	Positive	Positive	Positive	Negative
29.	Negative	Positive	Negative	Positive	Positive	Positive	Negative
30.	Negative	Positive	Negative	Positive	Positive	Positive	Negative
31.	Positive	Positive	Positive	Positive	Positive	Positive	Positive
32.	Positive	Positive	Positive	Positive	Positive	Positive	Positive
33.	Positive	Positive	Positive	Positive	Positive	Positive	Positive
34.	Positive	Positive	Positive	Positive	Positive	Positive	Positive
35.	Positive	Positive	Positive	Positive	Positive	Positive	Positive
36.	Positive	Positive	Positive	Positive	Positive	Positive	**Negative***
37.	Positive	Positive	Positive	Positive	Positive	Positive	Positive
38.	Positive	Positive	Positive	Positive	Positive	Positive	Positive

39.	Negative	Negative	Negative	Negative	Negative	Negative	Negative
40.	Negative	Negative	Negative	Negative	Negative	Negative	Negative
41.	Negative	Negative	Negative	Negative	Negative	Negative	Negative
42.	Negative	Negative	Negative	Negative	Negative	Negative	Negative
43.	Negative	Negative	Negative	Negative	Negative	Negative	Negative
44.	Negative	Negative	Negative	Negative	Negative	Negative	Negative
45.	Positive	Negative	Positive	Negative	NA	NA	Positive
46.	Positive	Negative	Positive	Negative	NA	NA	Positive
47.	Positive	Negative	Positive	Negative	NA	NA	Positive
48.	Positive	Negative	Positive	Negative	NA	NA	Positive
49.	Positive	Negative	Positive	Negative	NA	NA	Positive
50.	Positive	Negative	Positive	Negative	NA	NA	Positive
51.	Positive	Negative	Positive	Negative	NA	NA	Positive
52.	Positive	Negative	Positive	Negative	NA	NA	Positive
53.	Positive	Negative	Positive	Negative	NA	NA	Positive
54.	Positive	Negative	Positive	Negative	NA	NA	Positive
55.	Positive	Negative	Positive	Negative	NA	NA	Positive
56.	Positive	Negative	Positive	Negative	NA	NA	Positive
57.	Positive	Negative	Positive	Negative	NA	NA	Positive
58.	Positive	Negative	Positive	Negative	NA	NA	Positive
59.	Positive	Negative	Positive	Negative	NA	NA	Positive
60.	Positive	Negative	Positive	Negative	NA	NA	Positive
61.	Positive	Negative	Positive	Negative	NA	NA	Positive
62.	Positive	Negative	Positive	Negative	NA	NA	Positive
63.	Positive	Negative	Positive	Negative	NA	NA	Positive
64.	Positive	Negative	Positive	Negative	NA	NA	Positive
65.	Positive	Negative	Positive	Negative	NA	NA	Positive
66.	Positive	Negative	Positive	Negative	NA	NA	Positive
67.	Positive	Negative	Positive	Negative	NA	NA	Positive
68.	Positive	Negative	Positive	Negative	NA	NA	Positive
69.	Positive	Negative	Positive	Negative	NA	NA	Positive
70.	Positive	Negative	Positive	Negative	NA	NA	Positive
71.	Positive	Negative	Positive	Negative	NA	NA	Positive
72.	Positive	Negative	Positive	Negative	NA	NA	Positive
73.	Positive	Negative	Positive	Negative	NA	NA	Positive
74.	Positive	Negative	Positive	Negative	NA	NA	Positive
75.	Positive	Negative	Positive	Negative	NA	NA	Positive
76.	Positive	Negative	Positive	Negative	NA	NA	Positive
77.	Positive	Negative	Positive	Negative	NA	NA	Positive
78.	Positive	Negative	Positive	Negative	NA	NA	Positive
79.	Positive	Negative	Positive	Negative	NA	NA	Positive
80.	Positive	Negative	Positive	Negative	NA	NA	Positive
81.	Positive	Negative	Positive	Negative	NA	NA	Positive
82.	Positive	Negative	Positive	Negative	NA	NA	Positive

83.	Negative	Negative	Negative	Negative	NA	NA	Negative
84.	Negative	Negative	Negative	Negative	NA	NA	Negative
85.	Negative	Negative	Negative	Negative	NA	NA	Negative
86.	Negative	Negative	Negative	Negative	NA	NA	Negative
87.	Negative	Negative	Negative	Negative	NA	NA	Negative
88.	Negative	Negative	Negative	Negative	NA	NA	Negative
89.	Positive	Negative	Positive	Negative	NA	NA	NA
90.	Positive	Negative	Positive	Negative	NA	NA	NA
91.	Positive	Negative	Positive	Negative	NA	NA	NA
92.	Positive	Negative	Positive	Negative	NA	NA	NA
93.	Positive	Negative	Positive	Negative	NA	NA	NA
94.	Positive	Negative	Positive	Negative	NA	NA	NA
95.	Positive	Negative	Positive	Negative	NA	NA	NA
96.	Positive	Negative	Positive	Negative	NA	NA	NA
97.	Positive	Negative	Positive	Negative	NA	NA	NA
98.	Positive	Negative	Positive	Negative	NA	NA	NA
99.	Positive	Negative	Positive	Negative	NA	NA	NA
100.	Positive	Negative	Positive	Negative	NA	NA	NA
101.	Positive	Negative	Positive	Negative	NA	NA	NA
102.	Positive	Negative	Positive	Negative	NA	NA	NA
103.	Positive	Negative	Positive	Negative	NA	NA	NA
104.	Positive	Negative	Positive	Negative	NA	NA	NA
105.	Positive	Negative	Positive	Negative	NA	NA	NA
106.	Positive	Negative	Positive	Negative	NA	NA	NA
107.	Positive	Negative	Positive	Negative	NA	NA	NA
108.	Positive	Negative	Positive	Negative	NA	NA	NA
109.	Positive	Negative	Positive	Negative	NA	NA	NA
110.	Positive	Negative	Positive	Negative	NA	NA	NA
111.	Positive	Negative	Positive	Negative	NA	NA	NA
112.	Positive	Negative	Positive	Negative	NA	NA	NA
113.	Positive	Negative	Positive	Negative	NA	NA	NA
114.	Positive	Negative	Positive	Negative	NA	NA	NA
115.	Positive	Negative	Positive	Negative	NA	NA	NA
116.	Positive	Negative	Positive	Negative	NA	NA	NA
117.	Positive	Negative	Positive	Negative	NA	NA	NA
118.	Positive	Negative	Positive	Negative	NA	NA	NA
119.	Positive	Negative	Positive	Negative	NA	NA	NA
120.	Positive	Negative	Positive	Negative	NA	NA	NA
121.	Positive	Negative	Positive	Negative	NA	NA	NA
122.	Positive	Negative	Positive	Negative	NA	NA	NA
123.	Positive	Negative	Positive	Negative	NA	NA	NA
124.	Positive	Negative	Positive	Negative	NA	NA	NA
125.	Positive	Negative	Positive	Negative	NA	NA	NA
126.	Positive	Negative	Positive	Negative	NA	NA	NA

127.	Positive	Negative	Positive	Negative	NA	NA	NA
128.	Positive	Negative	Positive	Negative	NA	NA	NA
129.	Positive	Negative	Positive	Negative	NA	NA	NA
130.	Positive	Negative	Positive	Negative	NA	NA	NA
131.	Positive	Negative	Positive	Negative	NA	NA	NA
132.	Positive	Negative	Positive	Negative	NA	NA	NA
133.	Positive	Negative	Positive	Negative	NA	NA	NA
134.	Positive	Negative	Positive	Negative	NA	NA	NA
135.	Positive	Negative	Positive	Negative	NA	NA	NA
136.	Positive	Negative	Positive	Negative	NA	NA	NA
137.	Positive	Negative	Positive	Negative	NA	NA	NA
138.	Positive	Negative	Positive	Negative	NA	NA	NA
139.	Positive	Negative	Positive	Negative	NA	NA	NA
140.	Positive	Negative	Positive	Negative	NA	NA	NA
141.	Positive	Negative	Positive	Negative	NA	NA	NA
142.	Positive	Negative	Positive	Negative	NA	NA	NA
143.	Positive	Negative	Positive	Negative	NA	NA	NA
144.	Positive	Negative	Positive	Negative	NA	NA	NA
145.	Positive	Negative	Positive	Negative	NA	NA	NA
146.	Positive	Negative	Positive	Negative	NA	NA	NA
147.	Positive	Negative	Positive	Negative	NA	NA	NA
148.	Positive	Negative	Positive	Negative	NA	NA	NA
149.	Positive	Negative	Positive	Negative	NA	NA	NA
150.	Positive	Negative	Positive	Negative	NA	NA	NA
151.	Negative	Positive	Negative	Positive	NA	NA	NA
152.	Negative	Positive	Negative	Positive	NA	NA	NA
153.	Negative	Positive	Negative	Positive	NA	NA	NA
154.	Negative	Positive	Negative	Positive	NA	NA	NA
155.	Negative	Positive	Negative	Positive	NA	NA	NA
156.	Negative	Positive	Negative	Positive	NA	NA	NA
157.	Negative	Positive	Negative	Positive	NA	NA	NA
158.	Positive	Positive	Positive	Positive	NA	NA	NA
159.	Positive	Positive	Positive	Positive	NA	NA	NA
160.	Positive	Positive	Positive	Positive	NA	NA	NA
161.	Positive	Positive	Positive	Positive	NA	NA	NA
162.	Negative	Negative	Negative	Negative	NA	NA	NA
163.	Negative	Negative	Negative	Negative	NA	NA	NA
164.	Negative	Negative	Negative	Negative	NA	NA	NA
165.	Negative	Negative	Negative	Negative	NA	NA	NA
166.	Negative	Negative	Negative	Negative	NA	NA	NA
167.	Negative	Negative	Negative	Negative	NA	NA	NA
168.	Negative	Negative	Negative	Negative	NA	NA	NA
169.	Negative	Negative	Negative	Negative	NA	NA	NA
170.	Negative	Negative	Negative	Negative	NA	NA	NA

171	Negative	Negative	Negative	Negative	NA	NA	NA
172.	Negative	Negative	Negative	Negative	NA	NA	NA
173.	Negative	Negative	Negative	Negative	NA	NA	NA
174.	Negative	Negative	Negative	Negative	NA	NA	NA
175.	Negative	Negative	Negative	Negative	NA	NA	NA
176.	Negative	Negative	Negative	Negative	NA	NA	NA
177.	Negative	Negative	Negative	Negative	NA	NA	NA
178.	Negative	Negative	Negative	Negative	NA	NA	NA
179.	Negative	Negative	Negative	Negative	NA	NA	NA
180.	Negative	Negative	Negative	Negative	NA	NA	NA
181.	Negative	Negative	Negative	Negative	NA	NA	NA
182.	Negative	Negative	Negative	Negative	NA	NA	NA
183.	Negative	Negative	Negative	Negative	NA	NA	NA
184.	Negative	Negative	Negative	Negative	NA	NA	NA
185.	Negative	Negative	Negative	Negative	NA	NA	NA
186.	Negative	Negative	Negative	Negative	NA	NA	NA
187.	Negative	Negative	Negative	Negative	NA	NA	NA
188.	Negative	Negative	Negative	Negative	NA	NA	NA
189.	Negative	Negative	Negative	Negative	NA	NA	NA
190.	Negative	Negative	Negative	Negative	NA	NA	NA
191.	Negative	Negative	Negative	Negative	NA	NA	NA
192.	Negative	Negative	Negative	Negative	NA	NA	NA
193.	Negative	Negative	Negative	Negative	NA	NA	NA
194.	Negative	Negative	Negative	Negative	NA	NA	NA
195.	Negative	Negative	Negative	Negative	NA	NA	NA
196.	Negative	Negative	Negative	Negative	NA	NA	NA
197.	Negative	Negative	Negative	Negative	NA	NA	NA
198.	Negative	Negative	Negative	Negative	NA	NA	NA
199.	Negative	Negative	Negative	Negative	NA	NA	NA
200.	Negative	Negative	Negative	Negative	NA	NA	NA
201.	Negative	Negative	Negative	Negative	NA	NA	NA
201.	Negative	Negative	Negative	Negative	NA	NA	NA
203.	Negative	Negative	Negative	Negative	NA	NA	NA
204.	Negative	Negative	Negative	Negative	NA	NA	NA
205.	Negative	Negative	Negative	Negative	NA	NA	NA
206.	Negative	Negative	Negative	Negative	NA	NA	NA
207.	Negative	Negative	Negative	Negative	NA	NA	NA
208.	Negative	Negative	Negative	Negative	NA	NA	NA
209.	Negative	Negative	Negative	Negative	NA	NA	NA
210.	Negative	Negative	Negative	Negative	NA	NA	NA
211.	Negative	Negative	Negative	Negative	NA	NA	NA
212.	Negative	Negative	Negative	Negative	NA	NA	NA
213.	Negative	Negative	Negative	Negative	NA	NA	NA
214.	Negative	Negative	Negative	Negative	NA	NA	NA

215.	Negative	Negative	Negative	Negative	NA	NA	NA
216.	Negative	Negative	Negative	Negative	NA	NA	NA

[@]Two tube LAMP assay Case 1 to case 216.
[#]Loopamp^{TM} Pan kit Case 1 to case 44
[$]Loopamp^{TM} Pf kit Case 1 to case 44
[^]Loopamp^{TM} Pv kit Case 1 to case 88

Positive- positive amplification of DNA with green fluorescence; Negative- no amplification of DNA retaining vial with orange color; NA- test not performed; Pf- *Plasmodium falciparum*; Pv- *Plasmodium vivax*, Pan- *Plasmodium* genus specific. *Discordant result observed.

**Color code**	**Description of sample**
Positive	*Plasmodium* genus positive by Loopamp Pan
Negative	True malaria negative samples (67)
Positive	True *P. vivax* positive samples (115)
Positive	True *P. falciparum* positive samples (22)
Positive	True cases of mixed infection (12)

Figure S1: Prototypical STARD diagram to report flow of participants through the study for Two-tube Pv/Pf LAMP assay

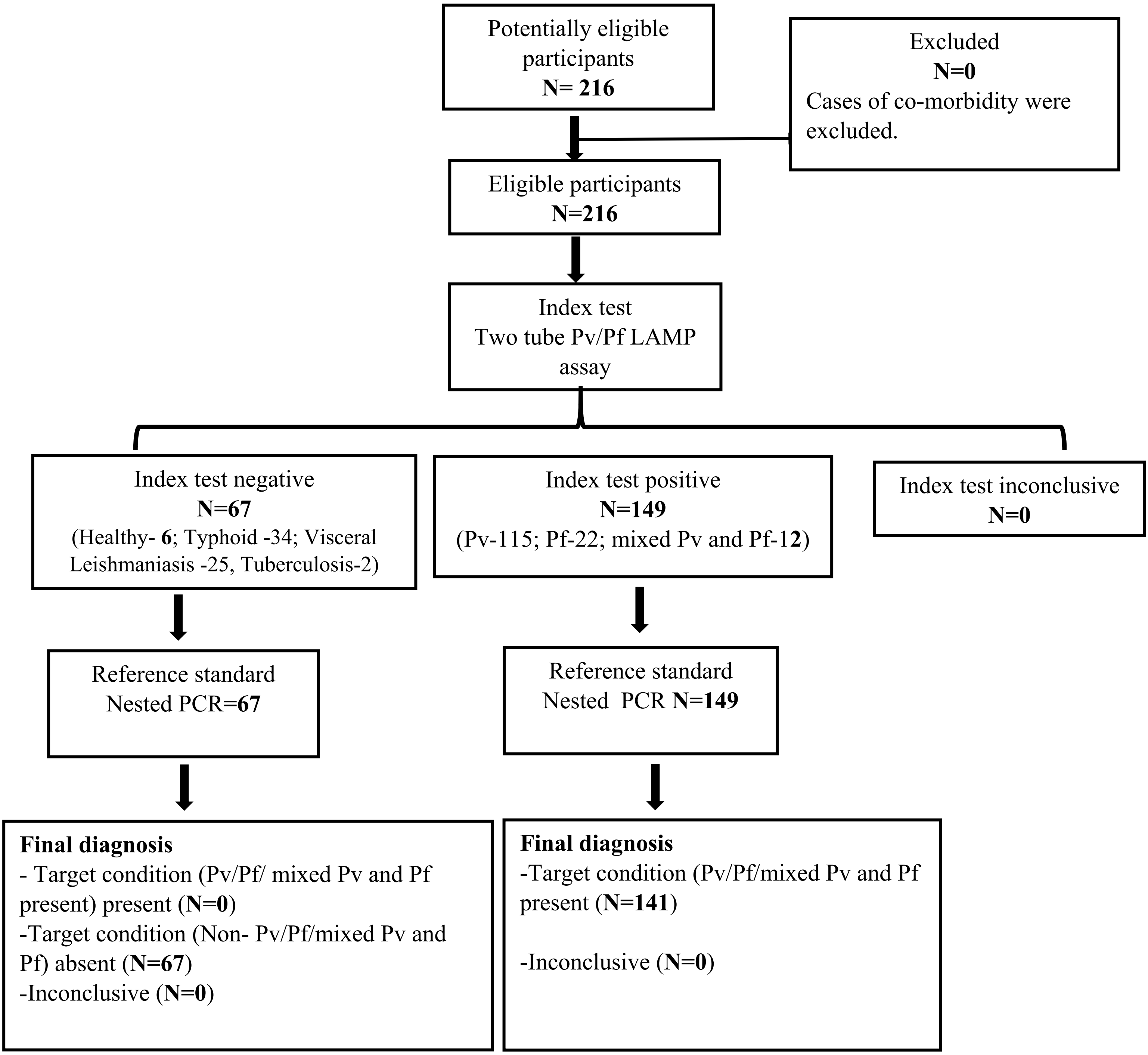

Figure S2: Prototypical STARD diagram to report flow of participants through the study for Loopamp™ Pan/Pf kit

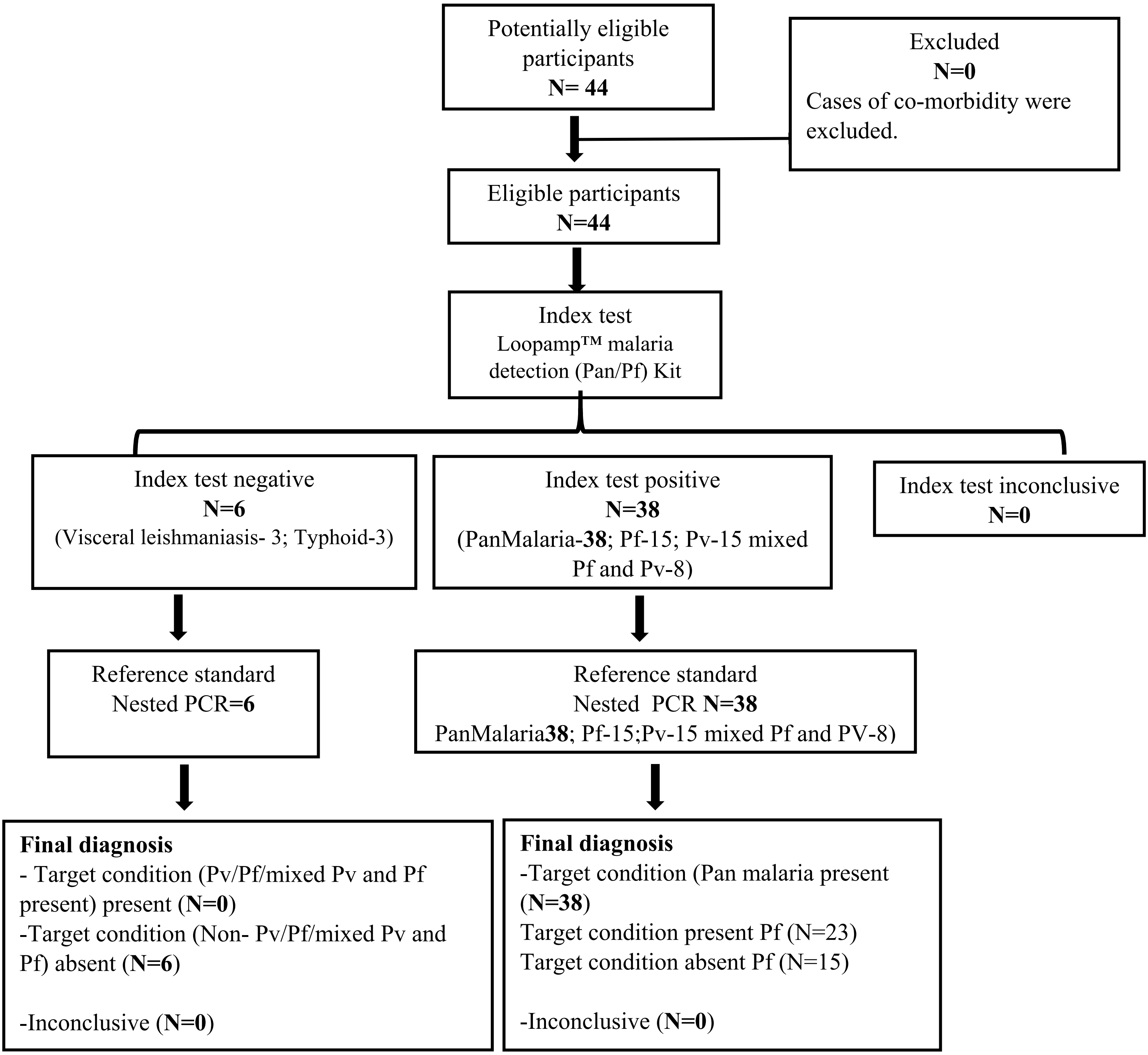

Figure S3: Prototypical STARD diagram to report flow of participants through the study Loopamp™ Pv kit

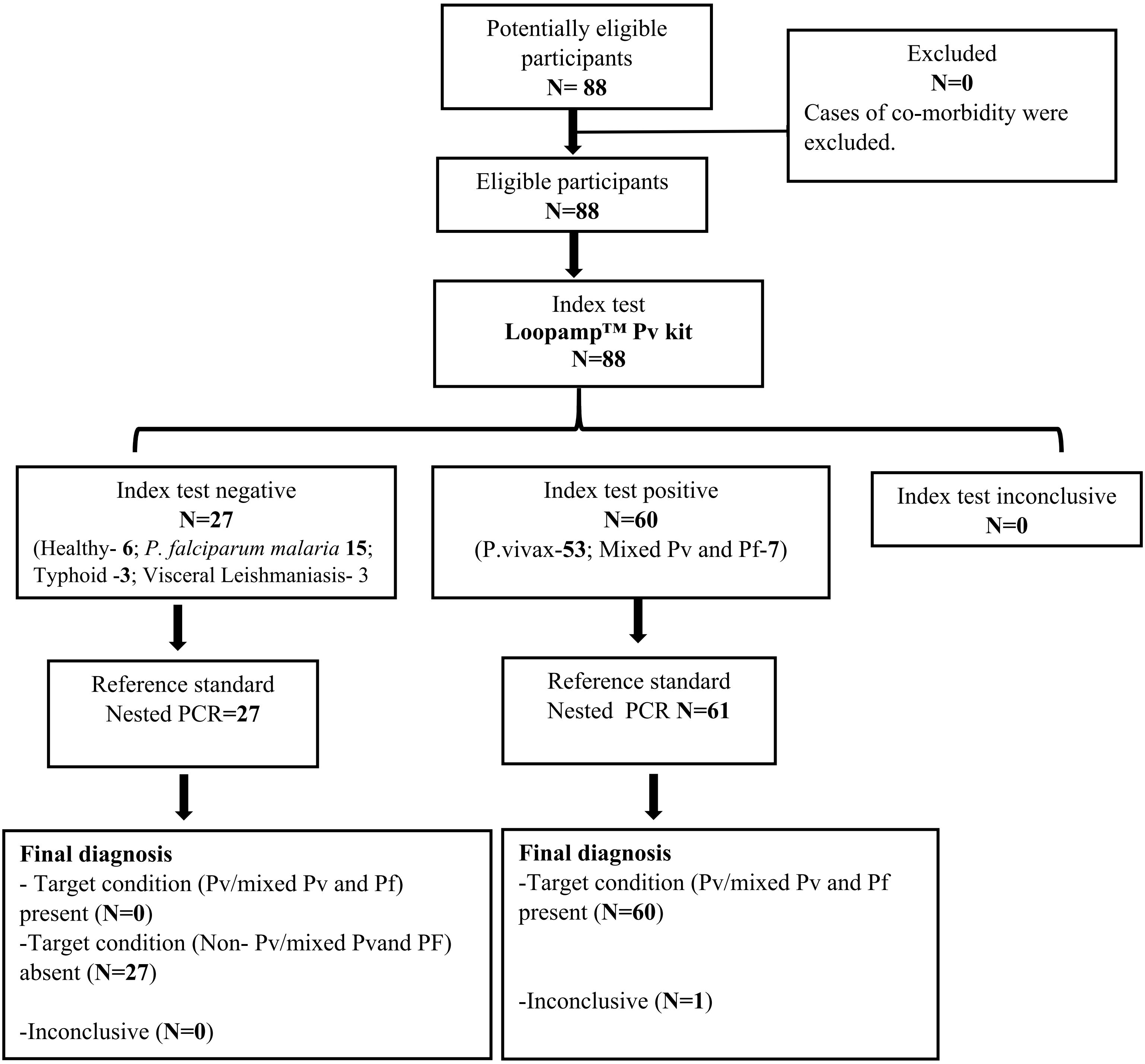

Supplement: Supplementary file 1 [file diagnostics-11-01689-s001.zip › diagnostics-1224998-supplementary.pdf]
